# Wireless Capsule Endoscope Localization with Phase Detection Algorithm and Adaptive Body Model

**DOI:** 10.3390/s22062200

**Published:** 2022-03-11

**Authors:** Paweł Oleksy, Łukasz Januszkiewicz

**Affiliations:** Institute of Electronics, Lodz University of Technology, Politechniki 10 Street, 93-590 Lodz, Poland; lukasz.januszkiewicz@p.lodz.pl

**Keywords:** wireless endoscopes, human body models, wireless localization, FDTD, WBAN

## Abstract

Wireless capsule endoscopes take and send photos of the human digestive tract, which are used for medical diagnosis. The capsule’s location enables exact identification of the regions with lesions. This can be carried out by analyzing the parameters of the electromagnetic wave received from the capsule. Because the human body is a complex heterogeneous environment that impacts the propagation of wireless signals, determining the distance between the transmitter and the receiver based on the received power level is challenging. An enhanced approach of identifying the location of endoscope capsules using a wireless signal phase detection algorithm is presented in this paper. For each capsule position, this technique uses adaptive estimation of human body model permittivity. This approach was tested using computer simulations in Remcom XFdtd software using a numerical, heterogeneous human body model, as well as measurements with physical phantom. The type of transmitting antenna employed in the capsule also has a significant impact on the suggested localization method’s accuracy. As a result, the helical antenna, which is smaller than the dipole, was chosen as the signal’s source. For both the numerical and physical phantom studies, the proposed technique with adaptive body model enhances localization accuracy by roughly 30%.

## 1. Introduction

Diagnosis of the gastrointestinal tract can now be performed with the use of capsule endoscopes, which provide doctors with a lot of valuable information. Such capsules are fitted with tiny cameras that allow them to detect anatomical changes without causing any discomfort to the patient. When using such endoscopes for medical examinations, the picture delivered from the capsule must be complemented by information regarding its precise location. It is critical for surgical or precise medication delivery in the therapy of the diagnosed disease. It is crucial to know not just where the capsule is located along the gastrointestinal system, but also where it is in three dimensions to correlate data from the capsule with results of other medical imaging techniques (e.g., magnetic resonance imaging—MRI). Many methods for locating endoscopic capsules have been presented [1,2,3,4,5,6,7,8,9,10,11,12,13,14,15,16,17], with the following approaches standing out: analysis of the picture captured by the endoscopic capsule [1,2,3,4,5,6], analysis of the electromagnetic field [7,8,9,10,11,12,13,14,15,16,17] and analysis and tracking of capsule movement using inertial sensors [4,14].

The detection of capsule location along the digestive tract can be carried out using image analysis methods. A motion tracking model is utilized in these systems, which is based on the analysis of characteristic features of subsequently recorded picture frames [1,2,3,4,5,6]. The objective is to detect and track the relevant feature point that is identical in two recorded frames. These points are assigned to different positions on the recorded frames as the capsule travels relative to them. This type of tracking necessitates the employment of an intestinal model to calculate the distance traveled by the capsule between two captured photos. As a result, the estimation accuracy in this technique is dependent on the parameters of the selected model as well as the quality of the recorded picture, and it may reach 86% on average. The speed of the capsules in the digestive tract limits image processing methods. The number of distinctive points in future frames collected by the camera reduces as the capsule’s speed rises. If the capsule speed is less than 4 mm/s, methods that estimate capsule speed can achieve accuracy of up to 93% [2].

There are also hybrid methods that employ data from a variety of sources or sensors to detect the capsule’s position and orientation in the digestive tract. The usage of a camera with a DSP (digital signal processor) to perform image processing algorithms is one example. It is used with a DMP (digital motion processor) in an inertial sensor to estimate the capsule’s orientation [3]. With the use of video-based approaches, this hybrid system can identify capsule position in three-dimensional space.

To find the location of the capsule in three dimensions, methods based on electromagnetic field analysis are applied. In a wireless capsule endoscope system, procedures based on electromagnetic field analysis can produce good results. The capsule was located with an accuracy of below 20 mm using an algorithm based on the distribution of the magnetic field [7,8]. Especially in a solution that uses a planar cable driven parallel robot (CDPR) and measurements of the quasistatic magnetic field of a Hall effect sensor, the localization error may be at the level of a few millimeters. In this method, by combining the hall sensor matrix—which is used to track permanent magnet movement in capsule—with the planar CDPR tracking control, the position of the capsule can be obtained by solving forward kinematics of CPRD [8]. This system is placed under the patient’s bed, which disqualifies the possibility of using this solution in wearable systems. In addition, taking into account the time of passage of the capsule through the digestive tract, proposed solution may require the patients to lie on their back for 8 h. Other methods use algorithms to estimate capsule position based on received signal parameters, such as power level, radio wave propagation delay or phase shift between the waves received by different receivers [9,10,11,12,13,14,15,16,17,18]. However, because of the complicated propagation environment within the body, converting received power or phase delay to the distance between the capsule and external receivers is problematic. It is caused by the human body’s complex heterogeneous structure, which has a significant impact on the received signal. Individual patient body models generated with the use of an MRI scanner can provide additional information that improves location accuracy. Unfortunately, due to the expense and time required for such scans, this strategy is unworkable. As a result, capsules employ inertial sensors and algorithms that may include digital filters devoted to movement tracking, such as particle or unscented Kalman filter (UKF) [3,11].

Algorithms based on phase detection can also be used to locate transmitters that are within the body. With the use of software-defined radio modules and RFID-enabled tags, such a system may be implemented in a realistic way that is both cost effective and provides useful item positioning precision [18]. A similar method may be used to locate an endoscope capsule; however, the complex environment of the human body makes such a device challenging to build due to wireless signal propagation effects. As a result, human tissue parameters, such as conductivity and dielectric permittivity, have a considerable impact on received signal parameters. Therefore, the authors suggested a new method in which a received signal phase detection algorithm is combined with a reduced human body model [17]. In the presented approach, the reduced human body model is a simplified human body model that, for each capsule location, approximates the body with homogenous material—the permittivity of which is adjusted for a particular location of the capsule. This model imitates only selected parameters or parts of the human body. In the case of the model used in the measurement experiment, the shape of the model and its internal structure were reduced. It was assumed that the model will have a size and shape similar to the human torso and electrical properties similar to the average value for the entire human body *ε_r_* = 52, which is commonly used in wearable system research.

In the case of capsule endoscope localization, the approach for verifying the localization algorithms is quite difficult and various verification setups were delivered to test proposed techniques. Received signal analysis is the most typical approach for verification based on computer simulations with numerical models of the human body [7,10,15,17]. Systems that use tracking and image processing are verified mainly with the images recorded by the endoscopes [1,2,3,4,5,6]. The next step is then the validation with the use of physical models and dedicated measuring devices. They can be made in the form of thin vessels filled with liquid simulating tissues and animal tissues, such as intestines. Robotic arms are typically used to imitate the capsule’s movement in such experiments. However, the validation of the performance of the algorithm for locating the capsule endoscope in the human digestive system is not possible. Such experiments that utilize prototype transmitters could be dangerous and, moreover, require the consent of the bioethical commission, which is not achievable at the initial stage of the research. For this reason, various physical phantoms are used with parameters and dimensions similar to the human body [16]. Another solution is to use animal tissues and organs [7], where the pig’s intestine can be used to verify the algorithms based on video analysis [3]. In the case of algorithms based on the analysis of the electromagnetic field, a heterogeneous numerical model of body is sufficient for verification because it allows taking into account phenomena such as a variation in the velocity of wave propagation and reflections and diffraction at the boundary of materials with different electrical parameters.

In this article, the research on wireless localization of endoscopic capsule that uses detection of the phase of the electromagnetic wave is presented. The results of the preliminary study on the algorithm were presented in [19], where the verification of simplified algorithm was performed with the use of computer simulations and heterogenous human body model in Remcom XFdtd software. Here, an enhanced version of the algorithm, in which the proposed function of localization error is minimized by iterative modification of model permittivity, is described. Compared to the previous version [19], it is supplemented now with additional calibration procedure that is required for localization based on measurement data. In this paper the measurement verification of localization algorithm is presented. Additionally, the antennas used in measurements are presented together with their impedance matching analysis.

The advantage of the proposed method over other techniques is that it does not require knowledge of the thickness and electrical parameters of individual tissues of the human body. That is why the suggested approach of wireless capsule localization has the benefit of being adaptable to patients of diverse body proportions. In contrast to the methods that use image analysis, it enables the localization of the endoscopic capsule in three-dimensional space. Therefore, the combination of these two methods makes it possible to locate capsule both along the digestive system and in the three-dimensional space.

## 2. Materials and Methods

Numerical models of the human body are widely used to design wireless systems that operate in this complex environment [20]. Depending on the exact application of such models, they differ in shape and internal structure [21]. They can be made of one material (homogeneous models) or from many materials of different electric properties (heterogeneous models). Additionally, the shape and size of models can be either identical with human body (anthropomorphic models) or different from this (simplified models). The last one mentioned can be used not only for the design of antennas and the analysis of the radio link in wearable systems but also for the localization of radio transmitters inside the body.

To estimate the velocity of wave propagation in the human body, the proposed localization technique employs a simplified body model. The wave velocity is mostly determined by the tissue relative permittivity, *ε_r_*, in the human body. The average value for the entire human body, *ε_r_* = 52, is commonly used in wearable system research, such as wearable antenna radiation pattern analysis. This approach is insufficient for endoscope capsule localization because local changes in body permittivity affect the phase of the wave received from the interior of the body. Average permittivity varies also depending on the anatomy of the patient in the thoracic area. Furthermore, because there are multiple tissues in the digestive tract with different permittivity values, the average value will fluctuate for each specific position of the capsule. Despite this, in the method that uses received signal analysis, the complex interaction of electromagnetic wave and the human body results in a particular phase shift. It can be interpreted, as the phase shift was not introduced by multi-tissue media but a uniform, homogenous material that is between two antennas. Such virtual material has its permittivity adjusted for a particular location of the endoscope in the body. This directly affects the distance determined from Equation (1).

Permittivity is the main feature of the implemented model, but in the proposed approach it has constant value for specified position of the capsule. In the proposed algorithm, the model has same value of permittivity towards each receiver, but it may be different for each capsule position. The algorithm implements a homogeneous model, which has an average permittivity value for a given capsule position, but model permittivity depends on the capsule position. As a consequence, in the suggested technique, the permittivity of a homogeneous model of the human body is automatically modified for each capsule position to increase the accuracy of the predicted position compared with findings achieved with constant permittivity.

**Figure 1:**
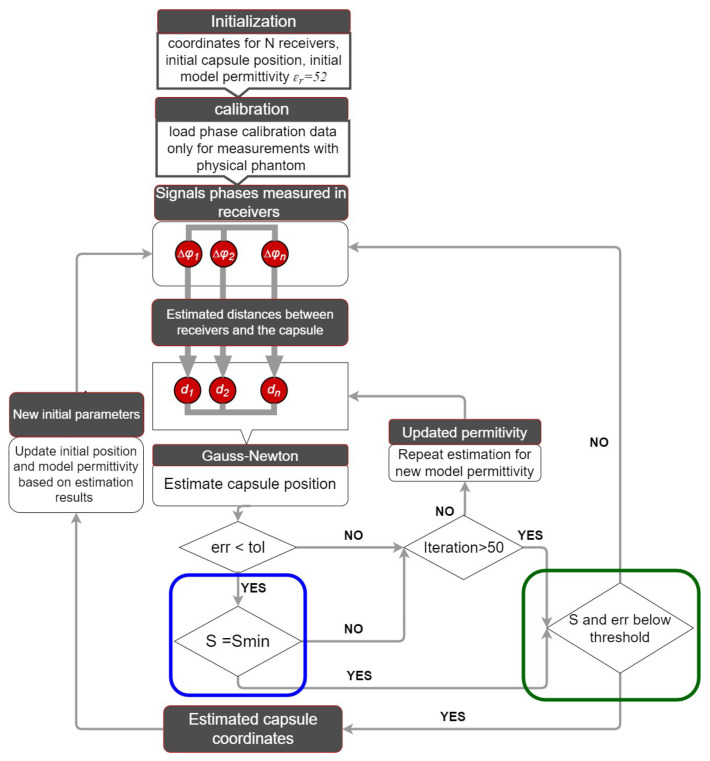
Improved localization algorithm with minimalization of *S* function marked in blue frame and results evaluation in green frame supplemented with calibration step for localization based on measurements.

The phase difference of arrival (PDoA) technique is used to determine capsule coordinates [10]. The phase difference of two transmitted signals with frequencies *f1* and *f2*, the coordinates of the *N*-th receiving antennas (shown in Figure 1), and the permittivity of a simplified human body model are all required for this procedure. The distances *d_n_* between the capsule and the receivers are determined in the first stage of the procedure (1). Because this necessitates knowledge of the model permittivity, the value *ɛ_r_* = 52 is assumed at the initial step. Because average permittivity towards each antenna may differ in a diverse environment, estimated distances *d_n_* (1) are only approximate.

(1) $$d_{n}=\frac{c}{2*\pi *\sqrt{\varepsilon_{r}}}*\frac{\Delta \varphi_{n}}{\Delta f}$$

*d_n_*—distance of the capsule from the *N*-th antenna placed on the human body; *c*—velocity of an electromagnetic wave in a vacuum; *ɛ_r_*—model permittivity; ∆*φ_n_*—phase difference of the received signals sent on two frequencies *f*1 and *f*2; ∆*f*—difference between frequency *f*1 and *f*2.

The Gauss–Newton algorithm is then used to estimate capsule coordinates, looking for coordinates (*u, v, x*) that allow the following function *S* to be minimized (2).

(2) $$S=\overset{N}{\underset{n=1}{\sum }}r_{n}^{2}$$

*N*—number of receiving antennas placed on the body,

$$r_{n}=d_{n}-\sqrt{{\left(x-u_{n}\right)}^{2}+{\left(y-v_{n}\right)}^{2}+{\left(z-w_{n}\right)}^{2}}$$

*x, y, z*—estimated capsule coordinates; *u_n_, v_n_, w_n_*—coordinates of antennas placed on the body.

The error function *err_i_* (3) is obtained for each estimated position. This represents the distance between the positions obtained in the last two iterations of the Gauss–Newton algorithm.

(3) $$err_{i}=\sqrt{{\left(x_{i}-x_{i-1}\right)}^{2}+{\left(y_{i}-y_{i-1}\right)}^{2}+{\left(z_{i}-z_{i-1}\right)}^{2}}$$

*err_i_*—error of the *i*-th iteration of the least squares method; *x_i_, y_i_, z_i_*—capsule coordinates estimated in the *i*-th iteration of the least squares algorithm, where 0 < *i* < 50.

The *err_i_* Function (3) was minimized using *ɛ_r_* that was modified for each iteration step in the initial version of the proposed algorithm [17]. The convergence criterion *err_i_* < 20 was not reached in that method for most capsule positions predicted for starting permittivity *ɛ_r_* = 52. For a transmitted signal frequency of 400 MHz, the difference between the final two approximations is bigger than the dimensions of a tuned dipole antenna.

The flowchart of an enhanced algorithm for capsule tracking inside the human body is shown in Figure 1. The initial permittivity is set to the average value for human tissue *ɛ_r_* = 52 at the starting point for the capsule, which is specified at the beginning of the digestive system. Verification of the algorithm performance in measurements with physical phantoms compared with the simulation [19] requires a calibration procedure at the beginning. The calibration data, which are the phase differences measured for each receiver placed directly at the capsule antenna, allow researchers to reduce the impact of the antennas construction, e.g., its case or cable connector on the localization accuracy. The capsule position is then determined using the Gauss–Newton technique based on recorded phase discrepancies. An additional step in the algorithm was included to increase the method’s accuracy. This minimizes the function *S*(2), according to Formula (4), by modifying the value of the model permittivity *ɛ_r_*, but only in the range for which the Gauss–Newton technique has achieved convergence in the previous phase. Practically, its value is in the range from 1 to 60.

(4) $$S_{min}=\underset{\varepsilon_{r}\;\epsilon \;<1,60>}{\min}|S(\varepsilon_{r})|$$

The proposed minimization procedure is an iterative process that can have up to 50 iterations or is repeated until the convergence criterion of Gauss–Newton is met. This allows finding the best value of model permittivity *ɛ_r_* for which the sum of squares of residuals described by *S* Functions (2) reaches its minimum value *S_min_* (4). The limit of iteration numbers in this step was introduced to prevent software deadlock caused by differences in tissue characteristics between the capsule and receiving antennas. Figure 1 shows the added step in the algorithm as a blue frame. When compared to the original version of the algorithm from [17], not only was the Gauss–Newton method’s convergence condition met, but the degree of accuracy of the *S*(2) function was also improved, implying that the difference between the distance computed with Equation (1), and the distance estimated by the Gauss–Newton method was reduced. The Gauss–Newton algorithm does not approach convergence for each measurement of the received signal phase due to the heterogeneous nature of the human body and the reflections of the electromagnetic wave. Because the phase of the received signal may change nonlinearly with frequency, the estimated distances to the following receiving antennas are ambiguous. The precision of the estimation obtained for the permittivity value for which the sum of the differences S(2) is the smallest is validated in the suggested technique by examining the values of the S and *err_i_* functions according to Formula (5) and (6). The value of the S function was assumed to be limited to S_th_ = N*r_n_^2^, where N denotes the number of external antennas and *r_n_* denotes the distance between capsule estimate and its real position.

In case of the simulation, the value *r_n_* was 30 mm and, respectively, for measurements to 150 mm, which is about 4 times the antenna’s maximum dimension. It was expected that the *err_i_* function’s result could not be more than the height of the intended antenna multiplied by four. The estimation found using this selection approach can be discarded in the tracking process, and the position will be revised based on new measurements. If the estimated position is affected by significant error (*S* and *err_i_* function above limits), then updating starting point may cause error propagation to the next estimation. Therefore, usage of determined values of the permittivity and position in next estimation depends on whether the *S* and *err_i_* functions can meet the described criteria. Figure 1 shows the introduced step of the algorithm in a green frame.

(5) $$S\left(\varepsilon_{r},\Delta \varphi \right)\;\leq \;N*r_{n}^{2}$$

*N*—number of receiving antennas; *r_n_*—distance between capsule estimate and its real position, constant value: 30 mm for simulation and 150 mm for measurements

(6) $$e_{rri}\left(\varepsilon_{r},\Delta \varphi \right)\;<\;4*H\;$$

*H*—helical antenna high used in simulation; practically the same value is also sufficient in case of localization based on measurement data.

It is feasible to adjust capsule position when new phase data become available to the algorithm. Although the variable parameter update in the first step has no effect on the final estimate, it does allow for a reduction in the number of algorithm iterations. With the data received for the latest successful estimation, the starting location and permittivity are updated. If the conditions in the green frame in Figure 1 are not satisfied, this step is skipped in order to prevent the mistake from propagating to the next location. If the conditions in the green frame (see Figure 1) are satisfied, then the capsule’s beginning position is updated with the predicted position.

## 3. Results

The algorithm’s verification consists of two steps. The verification in the first step was based on data gathered as from the computer simulation, whereas the data in the second stage came from measurements performed with simplified physical phantom. Computer simulations were obtained with Remcom XFdtd software version 7.8.1 [22] with the use of NMR heterogenous human body model with a 5 mm voxel size available in this tool. The set of 70 predefined positions of transmitting antenna in the area of the human digestive system was assumed in simulation scenario. The dimensions of the model are as follows: *lx* = 330 mm, *ly* = 290 mm, *lz* = 650 mm.

**Figure 2:**
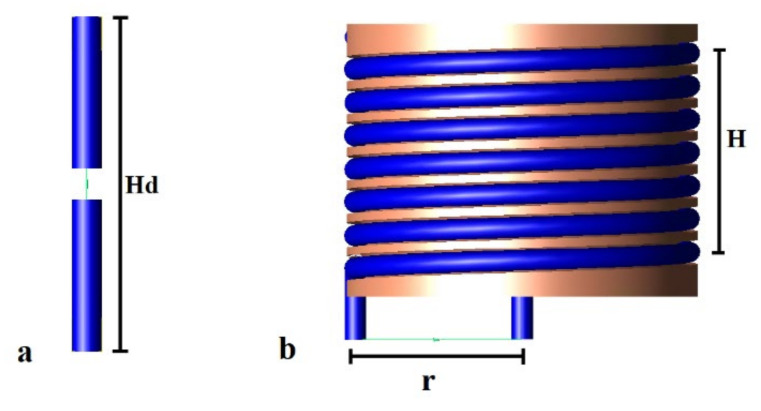
Transmitting antenna (**a**) half-wave dipole *Hd* = 22 mm, (**b**) helical antenna model: *H* = 5.6 mm, *r* = 4 mm [23].

Due to the size of the capsule endoscope (which is about 2 cm in longest dimension) and the size of the human gastrointestinal tract, it is desired to reduce antenna size as much as possible. On the other hand, the antenna size is limited by the signal frequency and is inversely proportional to it. In the case of capsule endoscopy, the frequency is strictly limited to the MICS band. The overall size of the antenna can be reduced by changing its structure, e.g., by forming the radiator in the shape of a spiral, yet maintaining satisfactory impedance matching. In the research presented here, we have focused on experimental verification of the algorithm. To perform the experiment, we had to fabricate small antennas that operate well in considered frequency band. Unlike endoscopic antennas, our experimental antennas had to be fed with coaxial cable that was connected to the network analyser. Due to this limitation, and considering the manufacturing technologies available in our laboratory, we decided to fabricate the antennas that were large enough to be soldered to coaxial probes. Since, in a real system, the antenna would be connected directly to the transceiver in the capsule, the antenna size was not the subject of our deep analysis here. Figure 2a [17] shows the half-wave dipole transmitting antenna utilized in the initial algorithm testing. The results in this article were obtained using the helical transmitting antennas shown in Figure 2b [23]. As external receivers, dipole antennas matched to 400 MHz in close proximity to the human body were utilized, which are the same as the dipole shown on Figure 2a [17].

**Figure 3:**
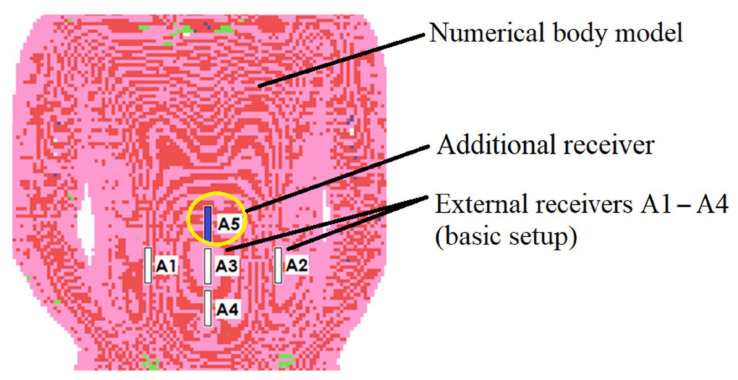
Arrangement of the receiving antennas A1–A5 relative to the numerical model used in simulation.

The receiving antennas were situated as near to the model as possible to reduce the influence of wave reflections, especially at the interface between the body model and the air. This resulted in antenna detuning; however, in the case of an external receiver, this was corrected by antenna length, which has no size limit in computer simulations. With the use of Equation (1), the placements of external antennas have been improved to reduce distance estimate inaccuracy. The antennas positioned on the torso in the front of the body gave the best accuracy [17]. In comparison to prior study, the addition of the extra receiver (A5 in Figure 3) enhanced localization accuracy. Figure 3 illustrates the simulation setup utilized in the algorithm’s numerical evaluation.

**Figure 4:**
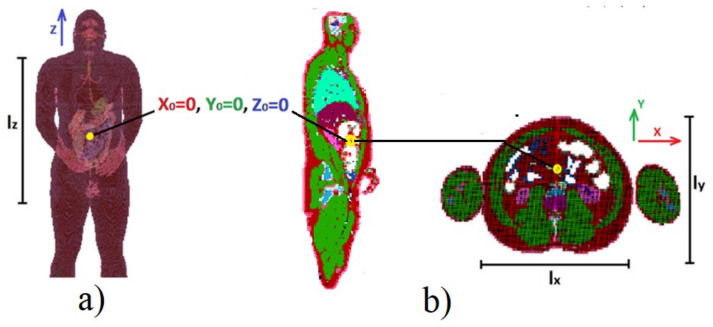
Numerical model used in simulations: (**a**) front view, (**b**) cross-section in XY plane for z = Z_0_ and for ZY for x = X_0_.

Because endoscopic capsules operate in the MICS band (from 401 MHz to 406 MHz), simulations for signals with frequencies of 403 MHz and 406 MHz were carried out [24]. Using the coordination system shown in Figure 4, the capsule location was modified in the range of x = 100 mm, y = 80 mm and z = 150 mm. The beginning of the coordinate system was regarded as the starting point for the Gauss–Newton localization algorithm.

The distance between the real and estimated positions of the transmitting antenna was defined as the localization error at a given point, which was defined by (7).

(7) $$Le=\sqrt{{\left(x_{t}-x_{est}\right)}^{2}+{\left(y_{t}-y_{est}\right)}^{2}+{\left(z_{t}-z_{est}\right)}^{2}}$$

*x_t_, y_t_, z_t_*—actual position; *x_est_, y_est_, z_est_*—coordinates of the estimated position.

The value of *S* can be minimized with model permittivity value. When examining the position estimate error, it was shown that both *S* and *Le* approach a minimum for similar values of *ɛ_r_* [19]. In this method the localization error can be reduced by decreasing the *S* function value. However, this method has a limitation resulting from the arrangement of external receivers. Reducing the location error in the proposed method is possible in about 80% of cases, as long as the capsule is located in the area limited in *x*- and *z*-axes by external receivers, but it grows considerably in other circumstances [19].

**Figure 5:**
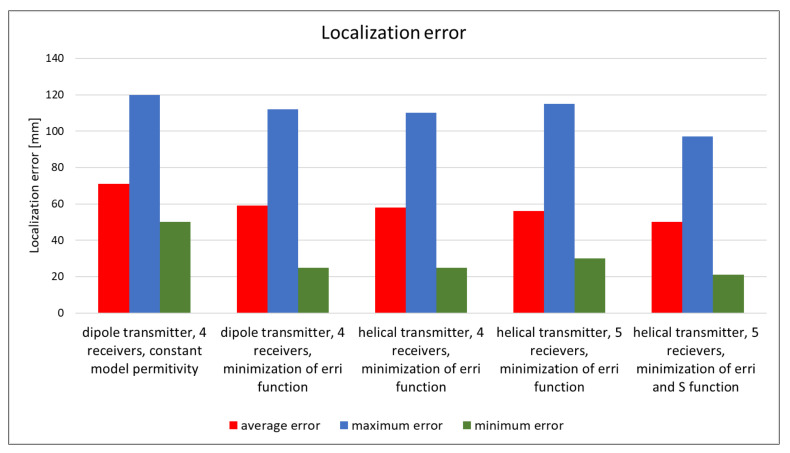
The impact of capsule antenna type, number of receivers and algorithm version on localization error achieved for simulation in XFdtd Remcom and heterogenous human body model [17,19].

The proposed localization method performance investigated in the simulation for different algorithm versions, transmitting antenna type and configuration of external receivers is presented on Figure 5. The solution with results validation shown in green frame on Figure 1 was used for each case. It can be noticed that both the introduction of the adaptive model of the human body, as well as the modification of the position of external receivers or the use of a different transmitting antenna, allows reduction in the localization error for the method which uses phase detection algorithm. When compared with a half-wave dipole, the helical antenna provides a relatively minor increase in location accuracy, but it has the benefit of being smaller, making it a better alternative for wireless endoscopes. Localization error also depends on the number of external receivers—in changing their number from 4 to 5, it is possible to improve the proposed method’s performance by a few percent.

**Figure 6:**
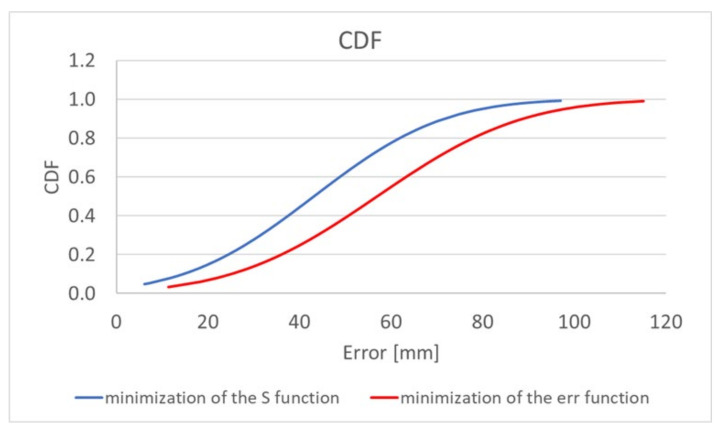
Cumulative distribution function (CDF) of localization error [19].

The algorithm, using the adaptive simplified model, also gives a significant improvement in localization accuracy. Minimization of both the *err_i_* and *S* functions allows a reduction in the mean error by about 30%; additionally, the minimum and maximum errors are reduced. The graph presented in Figure 6 [19] additionally shows the improvement that was achieved by extending the location algorithm by minimizing the S function comparing to its original version [17]. For the proposed antennas configuration and the adopted heterogeneous human body model, the localization error is mainly related to the *y*- and *z*-axes in accordance with the coordinate system shown in Figure 4 [19]. The simulations carried out showed that in the case of the *x*-axis the average localization error oscillates around 10 mm. This value is equivalent to the size of the endoscopic capsule (which in this case is 8 mm in diameter), thus it is deemed to be acceptable.

**Figure 7:**
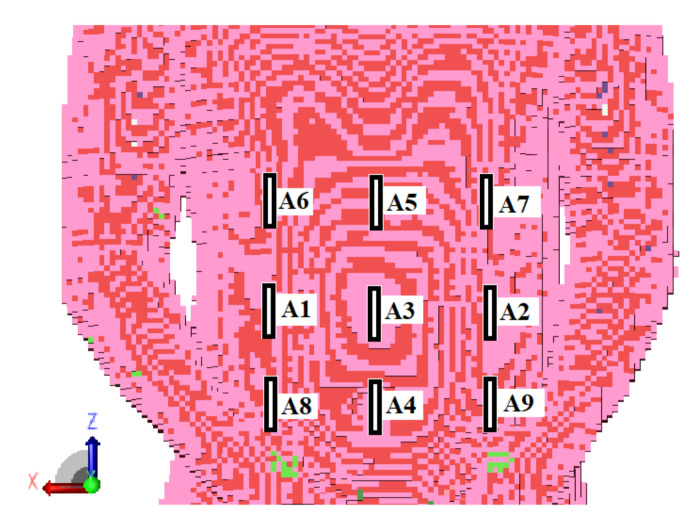
External receivers arrangement used to investigate the impact of the number of receivers on the accuracy of the location.

Considering the location error, the additional information of the signal phase coming from a larger number of receivers may not provide a significant improvement in the algorithm accuracy. Moreover, it may even increase the localization error. Table 1 shows the results of the capsule position estimation on the basis on information on the signal phase from nine external receivers arranged in in the form of a matrix, as presented in Figure 7. Presented results were obtained for 70 different capsule positions in the area of human digestive system. By introducing additional four receivers, the average localization error increased by about 9 mm.

**Figure 8:**
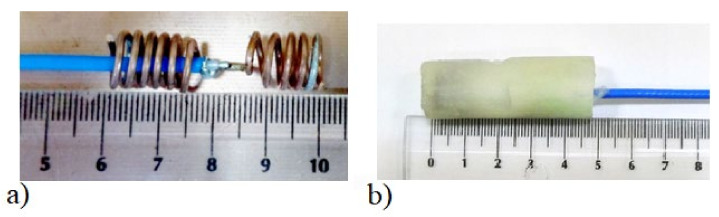
Capsule antenna that was located inside phantom (**a**) without case, (**b**) with epoxy case.

**Figure 9:**
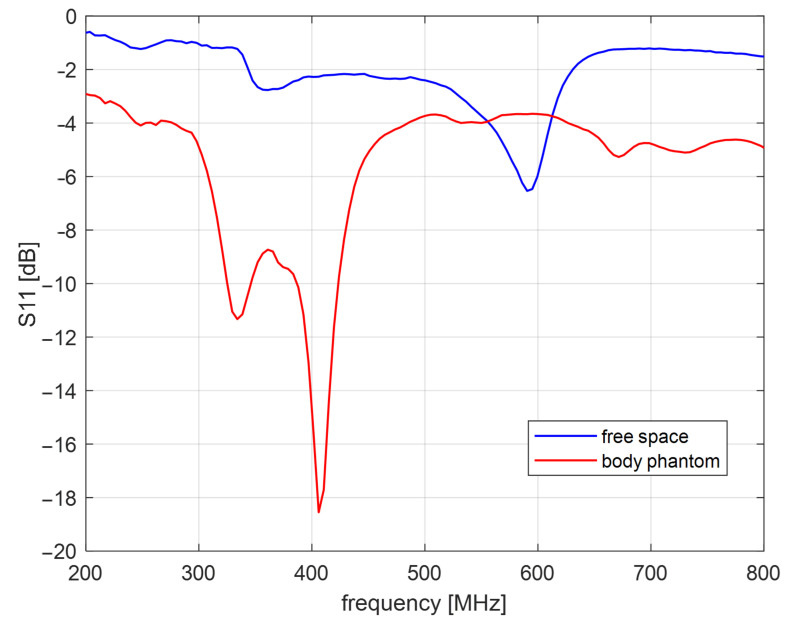
Impedance matching of the capsule antenna.

**Figure 10:**
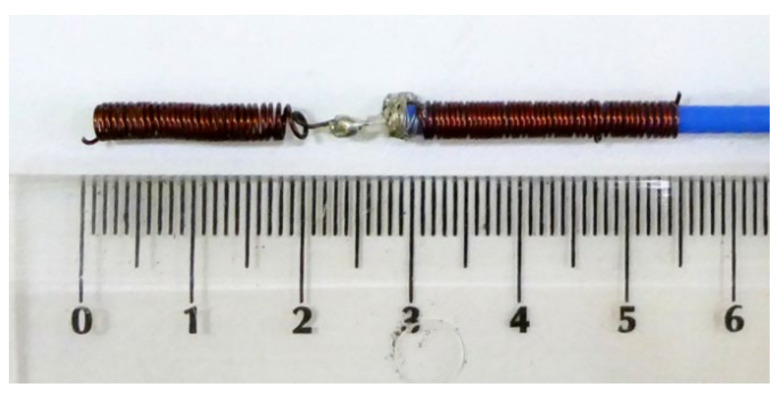
External receiver antenna.

**Figure 11:**
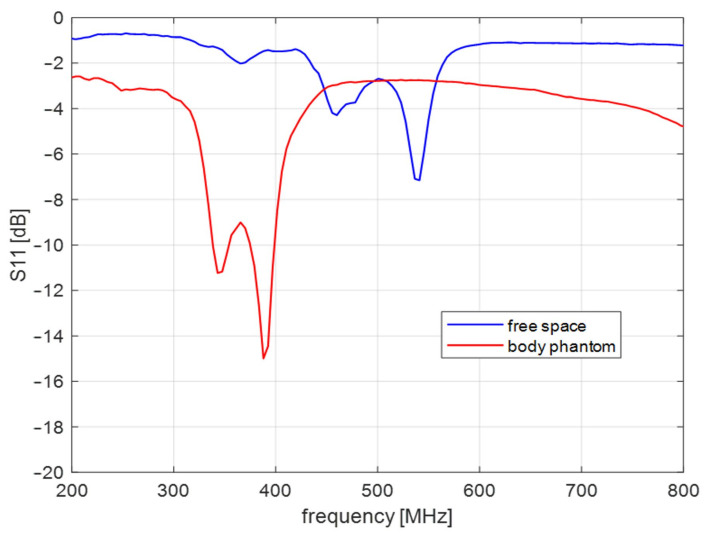
Impedance matching of the external receiver antenna.

Algorithm verification with measurement experiment was preceded with antennas and human body phantom design. Both external and capsule antennas are helical dipoles that were designed to obtain good impedance matching in MICS band. The proposed antenna used as capsule transmitter is presented in Figure 8. The transmitter antenna is placed in the tissue simulant liquid and requires the usage of additional case that limits the detuning effect. That is why the transmitting antenna (corresponding to the capsule endoscope) was surrounded by a case made of epoxy resin of relative permittivity 3.6 that is presented in Figure 8b. The impedance matching of this antenna is presented in Figure 9 for free space and location in tissue simulant liquid. It is well tunned for the 400 MHz band. The helical dipole for the external receiver it is presented in Figure 10. It was tuned to operate in desired frequency range being attached to the side of body phantom. Figure 11 shows that also this antenna is matched to MICS band for desired location.

**Figure 12:**
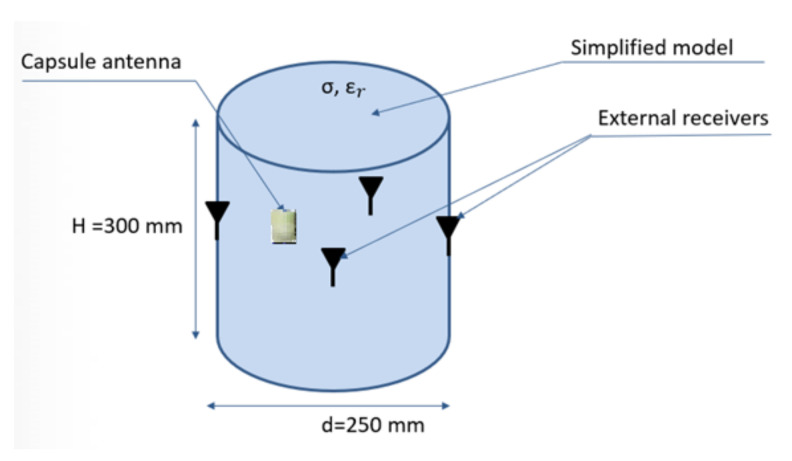
Simplified model and external antennas configuration used during measurement.

At this stage, it was assumed that physical phantom would be a homogeneous model made in the form of a thin-walled cylinder with a diameter of 25 cm and a height of 30 cm, as presented in Figure 12. For the purpose of this model, the measurements of electric properties of tissue simulating fluids were performed. Basing on data given in the literature, it was decided to use fluid with electrical parameters similar to the average parameters for the tissues of the human body. In the proposed experiment, it was assumed that the model parameters should be similar to the average values that are used in the case of electromagnetic simulation and experiments for wearable systems [20]. Electric parameters, as well as the proportions of the prepared solution, were defined experimentally to be as close as possible to permittivity *ɛ_r_* = 52 and the electrical conductivity *σ* = 1.8 S/m. For this sake, a solution of water, sugar and salt was prepared in the following proportions: 1 L water, 350 g sugar and 15 g salt. The exact values of permittivity and conductivity of prepared solution may vary additionally on the source of water and the frequency of interest. In the case of the water, the presence of additional minerals may change the parameters of the final solution. For this reason, both the permittivity and the conductivity of the prepared fluid were measured with DiLine measurement setup procedure by Index Sar Company. This uses TEM lines filled with liquid and vector network analyser that measures the change of electric length and loss of line [25]. The permittivity of tissue simulant solution, measured at the temperature of 23 °C at 400 MHz, was equal to *ε_r_* = 57, and the conductivity was *σ* = 0.75 S/m.

Measurements of phase difference between antennas were carried out with the use of Anritsu Vector Star MS4647B vector network analyzer (VNA), connected to capsule and external antennas via measurement cable. The calibration plane was placed at the connection point between the antenna and the measurement cable. Phase difference was measured as group delay, which is the rate of change of phase over a specified frequency aperture. In consequence Equation (1) will take the following form (8):

(8) $$d_{n}=\frac{c}{\sqrt{\varepsilon_{r}}}*tg$$

*d_n_*—distance of the capsule from the *N*-th antenna placed on the human body; *c*—velocity of light in a vacuum; *ɛ_r_*—model permittivity;$\;tg=\frac{1}{2\pi }\frac{\Delta \varphi_{n}}{\Delta f}$—group delay.

**Figure 13:**
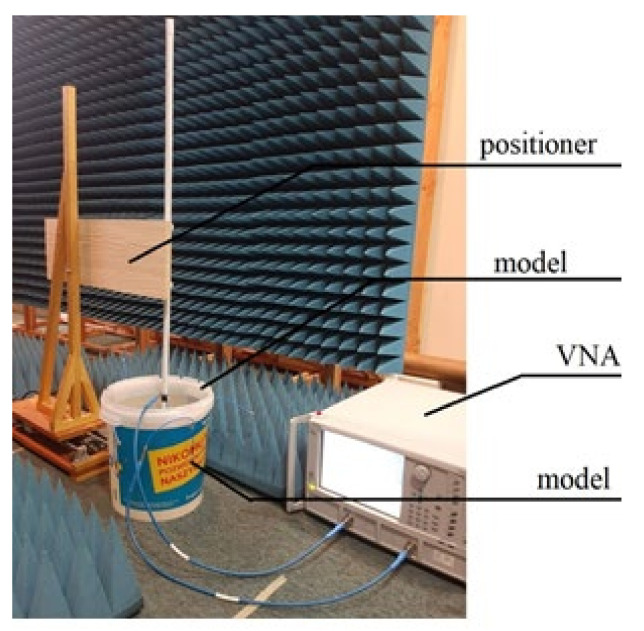
Measurement setup.

The capsule antenna was attached to the precise linear positioner controlled by computer. It allowed us to change the capsule position inside simplified model (Figure 13). A holder arm made of wood was used to minimize the positioner’s influence on the measurements. The computer application allows the capsule to be moved by a given distance. After setting the capsule in the given position, the measurement of group delay is performed.

There are some technical limitations of this positioner that had an impact on the experiment. It only allowed us to change the position of the capsule along one axis. For the purpose of the experiment, the position of the model was manually changed to change the direction of capsule path. Another limitation of this positioner is that it can only move with a constant speed, so that the speed of the capsule was about 2–3 cm/s, which corresponds to the average speed of a peristalsis wave.

In order to verify the error of the endoscopic capsule position estimation, it was necessary to perform a phase measurement for the position for which the actual geometrical coordinates of the capsule are known. For this reason, in the experiment, the capsule was stopped for the short time needed for the measurement, and then it was moved to the next position.

This additional material—the case of the capsule antenna—also impacts the measurement of the group delay in each of the external receivers. During measurements, we noticed that even when the capsule antenna is located directly next to the receiver, the measured group delay is relatively large. For example, the group delay measured directly at one of the receiving antennas was about 3.7 ns, while after the antenna was moved in a straight line by 25 cm, it rose to 4.7 ns. The difference in group delay in these two capsules’ positions is relatively small compared with the value measured directly by the receiver. Therefore, the calibration was applied to find the value of the group delay introduced in the antennas and connecting cables. Such calibration assumes that the antennas are close to each other, and that the delay introduced in the system comes from the antennas and not their separation. Another issue related with calibration is that it requires a known position of the antennas. In a practical case, this is possible when the capsule was just swallowed and is located in the beginning of the digestive tract. That is why we considered the closest distance between the antennas as a candidate solution for calibration procedure.

At the first stage of experiment, a calibration measurement was performed by measuring the group delay for each of the receivers placed successively directly at the capsule antenna. This data is loaded only once in the location algorithm at the beginning after the algorithm initialization procedure, as shown in Figure 1.

**Figure 14:**
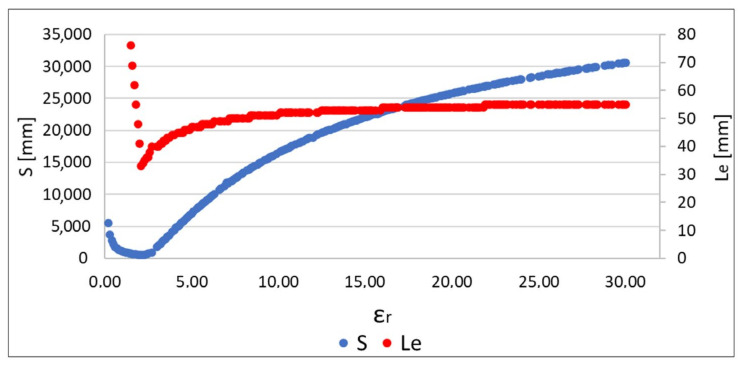
The value of *S* function and localization error (*Le*) depending on model permittivity.

Algorithm verification was also preceded by a check of whether the relationship between the value of the *S* function and the location error *Le* was similar to the observation for the data obtained as a result of the simulation [19]. It can be seen in Figure 14 that, for similar values of *ɛ_r_*, in both S and *Le* functions, it reaches a minimum. Therefore, the proposed algorithm can also be used for measurement data to reduce localization error.

**Figure 15:**
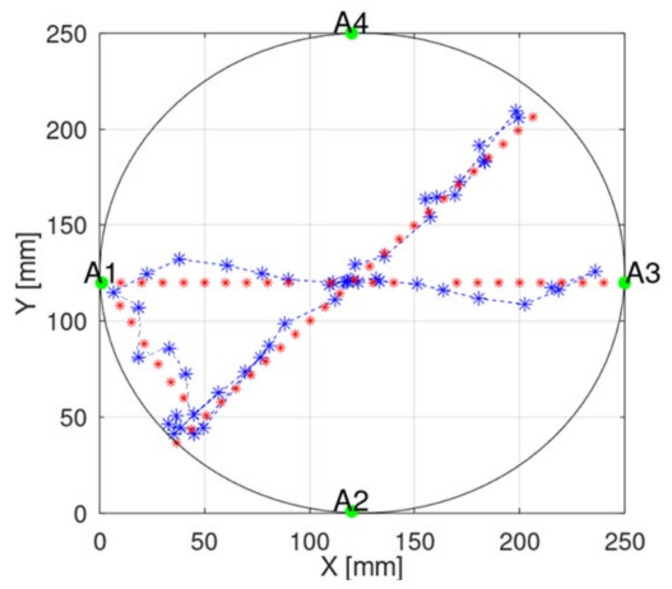
The example of implemented measurement scenario presented in the cross-section of a physical model. Red markers indicate true capsule positions, blue markers indicate estimated positions and A1–A4 indicate external receivers.

To illustrate the localization error in a better way, we added the presentation of the capsule position in a 2D plane to the manuscript. Figure 15 presents examples of capsule positions obtained with implemented measurements. Due to some positioners limitation, which allows for changing capsule position only on one axis, the capsule moved in straight lines. The main component of the overall estimation error is parallel to the capsule trajectory. This requires further investigation and probably the introduction of additional filtering techniques. Moreover, such an error may be also reduced by connecting the proposed algorithm with a video-based localization method, which gives very good results in cases of endoscopes localization along the digestive system.

**Figure 16:**
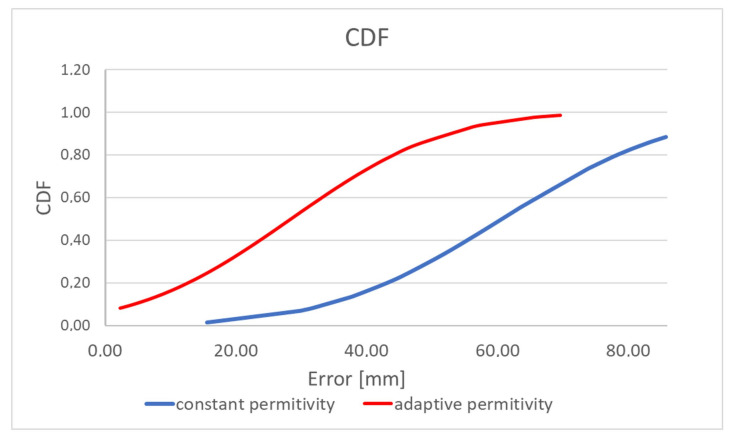
Cumulative distribution function (CDF) of localization error for measurements with physical phantom.

Table 2 and Figure 16 present the results of comparing the capsule endoscope localization algorithm with the constant and adaptive model permittivity. Because for the purpose of the experiment, a linear positioner was used with the antenna fixed to ground distance, the capsule position was estimated in 2D, changing its position in the x and y axes. In the case of an algorithm variant with constant permittivity, the value measured with TEM line *ε_r_* = 57 was applied. It can be noticed that average localization error for algorithm with adaptive model permittivity is reduced more than 2 times. Moreover, the algorithm was able to estimate the capsule position in 90% cases, while for the algorithm with constant model permittivity, it was only able to estimate the capsule position in 50% cases (cases for which the evaluation in Figure 1 in the green frame was positive). Permittivity estimated by the adaptive algorithm was in the range between 2 and 32. Thanks to this, it was possible to compensate for wave reflection in the phantom that impacts signal phase. For the algorithm with adaptive model permittivity, the localization error was only slightly bigger than the antenna size, so it can be assumed that it is at an acceptable level. Figure 15 shows that, for the experiment with the proposed model, the error is the largest along the capsule path.

**Figure 17:**
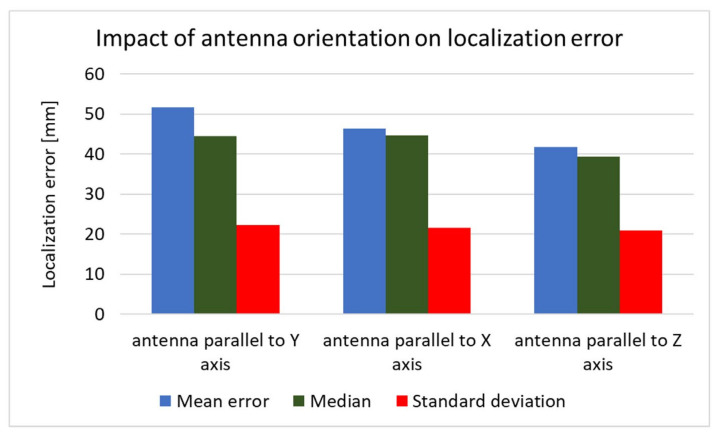
Impact of antenna orientation on localization error obtained in simulation.

**Figure 18:**
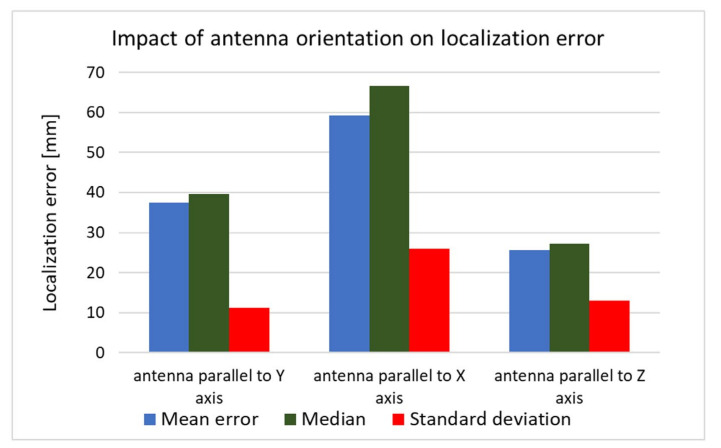
Impact of antenna orientation on localization error obtained in measurements.

The received signal is affected not only by the human body but also by the capsule’s orientation. To investigate the impact of the antenna orientation on localization accuracy, both simulation and measurement experiments were carried out. For this purpose, the capsule antenna was rotated by 90° around the *x* and *y* axes. Results presented in Figure 17 and Figure 18 show that, in the case of vertical orientation (antenna parallel to *z*-axis), the localization error was the smallest for both the measurement experiment and simulation.

The computational time of the algorithm and the overall response time of the whole tracking software in each iteration depends mainly on the error in estimating the distance between the capsule and the receivers at this particular location. It has a direct impact on the number of iterations of the Gauss–Newton algorithm. In the case of the simulation, the computation time of the single algorithm iteration was much shorter than in the case of the analysis of measurement data. This is because the Gauss–Newton algorithm achieved the convergence condition in a much smaller number of iterations. To estimate capsule position based on measurement, the algorithm requires around 8 times more steps than in case of simulation. The single position estimation time is equal in average to 0.18 s, while for measurements data, it is 1.52 s.

The proposed tracking software may be applied in two modes: offline and online. In offline mode, the phase difference or group delay are collected in a file, then after the experiment, the algorithm estimates the capsule path. In such an approach, the response time of the whole tracing software is the time needed for single position estimation multiplied by number of measurements. In the case of the experiment in which the capsule position was determined for about 70 cases, this time was, correspondingly, equal to 107 s in case the of measurements, and 12.6 s in the case of simulation data. In the online mode, the total tracking time depends on the speed of the capsule, assuming that the next measurement is performed immediately after the algorithm estimates the position for the previous one.

Practically, in capsule endoscopy, it is important to associate the recorded image with the position along the digestive tract and in 3D space. For this reason, it is not necessary to monitor the position of the endoscope in real time. The reconstruction of the capsule path may take place after the completed examination, just before the analysis of the collected data by the doctor.

## 4. Discussion

Upon the analysis of simulation and measurement results, it can be observed that the modified algorithm [19] gave better localization accuracy than the original algorithm [17]. The improvement in localization accuracy was observed in approximately 80% of cases when the *S* function was minimized by changing the value of model permittivity *ɛ_r_*. The findings in Figure 5 and Figure 6 demonstrate that the localization error has been decreased by roughly 15%. When the transmitter is inside the area confined by receivers in the *x* and *z* axes, the proposed technique, minimizing the *S* (2) function, provides an improvement.

Despite the fact that the presented version of the algorithm improves localization accuracy, estimation error is still higher than in methods based on magnetic field distribution analysis (below 2 cm) [7,8] or those based on the information of a patient’s body structure [13]. Methods based on magnetic field distribution give promising results in applications that require real-time tracking. On the other hand, these methods have some limitations in localization in three dimensions, resulting from the sensitivity of the magnetic field sensors. Moreover, such a solution cannot be applied in wearable systems. Algorithms that track capsules based on recorded pictures also provide acceptable accuracy, with an estimate error of less than 24.9 mm [3]. Most methods have limitations, such as capsule speed or digestive system models, that must be taken into account for each patient. The suggested adaptive body model technique has the benefit of being able to be employed for patients with a variety of body structures. The presented results show that an increased number of external receivers may cause an increase in estimation error. Additionally, the minimum of the *S* function and the minimum localization error *Le* may by achieved only if the capsule antenna is located in the area limited by the receiving antennas in the *x* and *z* axes. Therefore, development of an algorithm for dynamic selection of 4–5 receiving antennas from a given set might be a potential option.

Based on the simulation findings, it is evident that the type of transmitting antenna used in experiment has an impact on the algorithm’s accuracy, as well as the number of receivers. When a helical antenna and five receivers were used instead of a dipole transmitting antenna and four receivers, the localization error was decreased by around 9 mm.

## 5. Conclusions

In this paper, the improved method of wireless endoscope capsules localization with the use of phase detection algorithm and simplified human body model with adaptive permittivity was presented.

The suggested approach of wireless capsule localization has the benefit of being adaptable to patients with diverse body proportions. The main advantage of the proposed method is that it does not require knowledge of the internal structure and electrical parameters of individual tissues of the human body. The localization error is still higher than in methods that use information of electromagnetic field distribution, or in models created using NMR scanners that use the human body. The main contribution to the overall estimation error has an error component parallel to the capsule trajectory; therefore, combination of the proposed method, together with a video-based technique, may improve localization accuracy in 3D. The suggested method has a significant benefit in that it not only increases location accuracy, but it also allows researchers to filter out estimations with large errors. Experiments with physical models reveal that the suggested algorithm with the adaptive value of model permittivity not only improves location accuracy, but also allows researchers to reach convergence for a significantly greater number of capsule positions.

The verification of the algorithm with physical phantoms of the human body in three-dimensional coordinate systems, as well as multi-layered phantoms, will be the subject of future study. These tests will allow a final assessment of the suggested approach and the feasibility of its application in the study of the digestive system using wireless endoscopes.

## Figures and Tables

**Table 1:** The impact of the number of receivers on localization accuracy.

	Five Receivers and Adaptive Model	Nine Receivers and Adaptive Model	Five Receivers and Constant Permittivity
Average error (mm)	16	80	66
Standard Deviation (mm)	4	79	45
Meadian (mm)	2	67	40

**Table 2:** Localization accuracy for different algorithms.

	Minimum Error (mm)	Maximum Error (mm)	Average Error (mm)
Constant epsilon			
Localization error	16	80	66
Error for X	4	79	45
Error for Y	2	67	40
Adaptive model			
Localization error	3	62	30
Error for X	2	58	26
Error for Y	1	50	20

## Data Availability

The simulation results are available upon email request.
